# Identification of Metabolism-Associated Prostate Cancer Subtypes and Construction of a Prognostic Risk Model

**DOI:** 10.3389/fonc.2020.598801

**Published:** 2020-11-26

**Authors:** Yanlong Zhang, Ruiqiao Zhang, Fangzhi Liang, Liyun Zhang, Xuezhi Liang

**Affiliations:** ^1^ Department of Urology, First Hospital of Shanxi Medical University, Taiyuan, China; ^2^ First Clinical Medical College, Shanxi Medical University, Taiyuan, China; ^3^ Department of Rheumatology, Shanxi Bethune Hospital, Shanxi Academy of Medical Sciences, Taiyuan, China

**Keywords:** prostate cancer, metabolism-associated subtype, risk model, tumor heterogeneity, immunotherapy

## Abstract

**Background:**

Despite being the second most common tumor in men worldwide, the tumor metabolism-associated mechanisms of prostate cancer (PCa) remain unclear. Herein, this study aimed to investigate the metabolism-associated characteristics of PCa and to develop a metabolism-associated prognostic risk model for patients with PCa.

**Methods:**

The activity levels of PCa metabolic pathways were determined using mRNA expression profiling of The Cancer Genome Atlas Prostate Adenocarcinoma cohort *via* single-sample gene set enrichment analysis (ssGSEA). The analyzed samples were divided into three subtypes based on the partitioning around medication algorithm. Tumor characteristics of the subsets were then investigated using t-distributed stochastic neighbor embedding (t-SNE) analysis, differential analysis, Kaplan–Meier survival analysis, and GSEA. Finally, we developed and validated a metabolism-associated prognostic risk model using weighted gene co-expression network analysis, univariate Cox analysis, least absolute shrinkage and selection operator, and multivariate Cox analysis. Other cohorts (GSE54460, GSE70768, genotype-tissue expression, and International Cancer Genome Consortium) were utilized for external validation. Drug sensibility analysis was performed on Genomics of Drug Sensitivity in Cancer and GSE78220 datasets. In total, 1,039 samples and six cell lines were concluded in our work.

**Results:**

Three metabolism-associated clusters with significantly different characteristics in disease-free survival (DFS), clinical stage, stemness index, tumor microenvironment including stromal and immune cells, DNA mutation (*TP53* and *SPOP*), copy number variation, and microsatellite instability were identified in PCa. Eighty-four of the metabolism-associated module genes were narrowed to a six-gene signature associated with DFS, *CACNG4*, *SLC2A4*, *EPHX2*, *CA14*, *NUDT7*, and *ADH5* (p <0.05). A risk model was developed, and external validation revealed the strong robustness our risk model possessed in diagnosis and prognosis as well as the association with the cancer feature of drug sensitivity.

**Conclusions:**

The identified metabolism-associated subtypes reflected the pathogenesis, essential features, and heterogeneity of PCa tumors. Our metabolism-associated risk model may provide clinicians with predictive values for diagnosis, prognosis, and treatment guidance in patients with PCa.

## Introduction

Prostate cancer (PCa) is the second most frequent urinary system-associated type of cancer, accounting for 13% of all malignant tumors in men (1). Radical prostatectomy (RP) has been used to cure PCa patients by removing the malignant prostate. However, the recurrence rates after the surgery are high. Recurrent cancer has risks of developing into castration-resistant PCa, which will either continue progressing the pre-existing PCa or spreading cancer to other parts of the body (2). Therefore, exploring the tumor characteristic and finding a new therapy for PCa remains crucial. Furthermore, identifying biomarkers for disease-free survival (DFS) is needed to improve patients’ prognosis with PCa.

Due to the unrestricted multiplicative nature of cancer cells, tumors exhibit different metabolic statues from normal tissue, thus provide a possible way to identify tumors through the difference in metabolism. Recent studies have proven that some metabolisms, such as citrate and choline metabolism, are closely related to PCa (3). Studies have also shown that based on the variance in metabolites, such as increased urea cycle metabolites, PCa can be characterized (4).

Classification analysis based on a large number of samples that can better reflect tumor features and heterogeneity becomes possible with the advent of high-throughput sequencing. HIgh-throughput sequencing has been successfully applied to classify subtypes in different cancers. Subtypes are then used to either guide immune therapy, portray multiple dimensions of tumor characteristics, or assist patient prognosis prediction (5, 6). Although many genome-wide analyses have been performed in regards to PCa, there has been a lack of hierarchical cluster analyses of the PCa transcriptome to exploring tumor metabolic features. Meanwhile, almost all previous studies were based on PCa tumor metabolism concentrated on individual tumor cells rather than mixed tissue, including tumor cells, stromal cells, and immune cells. These studies and therefore do not reflect the metabolic characteristics of PCa *in vivo* (7). So a hierarchical cluster analysis of the PCa transcriptome from a metabolic view to exploring tumor heterogeneity is therefore crucial.

Based on the information above, we performed unsupervised clustering to explore the potential metabolism-associated subtypes and explored the correlations between the subtypes and tumor heterogeneity. Biomarkers associated with subtypes were also selected. Finally, a risk model to predict PCa patients’ prognosis was constructed. We hypothesize the metabolism-associated characteristics of PCa to understand the PCa metabolic mechanism better and further identify tumors. The risk model will be able to guide the PCa diagnosis, prognosis, and treatment.

## Materials and Methods

### Data Collection

Gene expression files, DNA mutation data, and copy number variation (CNV) of prostate adenocarcinoma (PRAD) tissues were downloaded from TCGA (https://portal.gdc.cancer.gov/). Gene expression data were acquired using the Illumina HiSeq RNA Sequencing platform and expressed as fragments per kilobase of transcript per million fragments (FPKM). The cBioPortal for Cancer Genomics (https://www.cbioportal.org/) provided clinical data of the PRAD patients (8). RNA sequencing (RNA-seq) data of normal prostate tissues from testing cohorts for diagnosis were obtained from the Genotype-Tissue Expression (GTEx) (https://www.gtexportal.org/) and tumor tissue from the ICGC (https://icgc.org/). RNA-seq and microarray data of PRAD tissues and clinical information from testing cohorts for prognosis were obtained from the Gene Expression Omnibus (GEO) database (https://www.ncbi.nlm.nih.gov/gds/). The GEO search strategy of the GSE datasets was as follows: 1) Include “prostate cancer” and dataset types of RNA-seq or micro-array; 2) Include more than one hundred PRAD samples with survival data; and 3) Include expression information of six risk model genes. Two datasets that met these requirements were identified, GSE54460 and GSE70768. Microarray data of cell lines (including 22RV1, DU-145, LNCaP-Clone-FGC, PC-3, PWR-1E, and VCaP), and RNA-seq data of melanoma samples were downloaded from the Genomics of Drug Sensitivity in Cancer (GDSC) (https://www.cancerrxgene.org/) and GSE78220. We also acquired the immunohistochemistry (IHC) data for PRAD and normal prostate tissues from the Human Protein Atlas (HPA) data portal (https://www.proteinatlas.org/). Immune infiltrate data for PRAD tissues were downloaded from the Cistrome Project (http://www.cistrome.org/) using the Tumor IMmune Estimation Resource version 2.0 (TIMER2.0) (9).

### ssGSEA Assessment of Metabolism-Associated Pathways Expression Levels

Data for 41 metabolism pathway gene sets were acquired from Molecular Signatures Database (MSigDB; https://www.gsea-msigdb.org/) (10) and Kyoto Encyclopedia of Genes and Genomes (KEGG) (11) and the PCa related activity levels were calculated using ssGSEA and the gene set variance analysis (GSVA) R package 1.34.0 (10). The metabolism-associated signatures used included galactose metabolism, ascorbate and aldarate metabolism, fatty acid metabolism, purine metabolism, pyrimidine metabolism, alanine aspartate and glutamate metabolism, glycine serine and threonine metabolism, cysteine and methionine metabolism, arginine and proline metabolism, histidine metabolism, tyrosine metabolism, phenylalanine metabolism, tryptophan metabolism, beta alanine metabolism, taurine and hypotaurine metabolism, selenoamino acid metabolism, glutathione metabolism, starch and sucrose metabolism, amino sugar and nucleotide sugar metabolism, glycerolipid metabolism, inositol phosphate metabolism, glycerophospholipid metabolism, ether lipid metabolism, arachidonic acid metabolism, linoleic acid metabolism, alpha linolenic acid metabolism, sphingolipid metabolism, pyruvate_metabolism, glyoxylate and dicarboxylate metabolism, propanoate metabolism, butanoate metabolism, riboflavin metabolism, nicotinate and nicotinamide metabolism, retinol metabolism, porphyrin and chlorophyll metabolism, nitrogen metabolism, sulfur metabolism, metabolism of xenobiotics by cytochrome P450, drug metabolism cytochrome P450, and drug metabolism other enzymes (**Table S1**).

### Identification of PRAD Subtypes by Partitioning Around Medication (PAM) and T-Distributed Stochastic Neighbor Embedding (t-SNE) Analyses

Unsupervised clustering analysis using the PAM algorithm was performed based on the ssGSEA score of each sample using the R package ConsensusClusterPlus function (12). The samples were then divided into three subtypes. The t-SNE analysis of the ssGSEA scores using R package Rtsne identified three clusters. Kaplan–Meier (K–M) survival analysis of the three metabolism-associated subtypes was performed using R package survival.

### Stemness Index Calculation and Immune Infiltration Estimation of PRAD Tumors

To evaluate the tumor stemness index, we downloaded the mRNA expression-based stemness index (mRNAsi) calculated by machine learning in previous studies (13). The stem cell gene set was obtained in a previous study (14), and the ssGSEA stemness index (ssGSEAsi) was calculated using the GSVA R package 1.34.0 (10). Tumor purity was calculated using R package ESTIMATE 1.0.13 (15) and then used to correct the stemness index. The immune scores, stromal scores, and ESTIMATION scores calculated using the R package ESTIMATE 1.0.13 (15) were used to evaluate immune cell and stromal cell abundance in the PRAD tumors.

### Metabolism-Associated Module Genes Filtered by WGCNA and Functional Enrichment Analysis

After selecting the metabolism-associated genes, we generated an adjacency matrix (AM) and topological overlap matrix (TOM) using the gradient method based on power values ranging from 1 to 20. When the correlation between the average degree of connectivity (k) and p (k) reached 0.88, we obtained the optimal power value and constructed a scale-free topology network. Network connectivity of the genes was measured using a TOM transformed from an AM (16). Modules were calculated using a divided cluster tree (17). Finally, we linked the module eigengenes (MEs) with the subtypes in the current study related to metabolism-associated status (C1, C2, and C3) and for the next analysis, selected the module with the highest correlation based on module-trait correlation coefficients and gene significance (GS) with C1 and C3 (ǀcorǀ >0.3). These genes were considered metabolism-associated module genes. To annotate the molecular functions of the genes, Gene Ontology (GO) and KEGG functional enrichment analyses of the metabolism-associated module genes were performed using the clusterProfiler R package 3.42.0 (18).

### Biomarker Selection and Risk Model Construction

The correlation between metabolism-associated genes and DFS of PRAD was calculated and analyzed using univariate Cox analysis with R package survival 3.1.8, and candidate biomarkers were screened at p values <0.05. LASSO regression using R package glmnet 3.0.2 (19, 20) was then applied to resolve any multilinear problem that may have existed in the regression analysis, and the biomarkers were filtered. Multivariate Cox was used next to build a risk model and to obtain estimated regression coefficients. Finally, we calculated the risk score for each sample to quantify the prognosis risk of each patient with PRAD. Survival data were analyzed as K–M survival curves *via* R package survival 3.1.8. To evaluate the precision of the risk model and nomogram, time-dependent receiver operating characteristic (ROC) analysis was applied using the R package survival ROC 1.0.3. An area under the ROC curve (AUC) >0.60. indicated the prediction ability of the model was meaningful, and an AUC >0.70 indicated an outstanding predictive value of the model. To investigate the function of risk model genes, we performed GSEA of the TCGA cohort according to the high-risk group and low-risk group divided by the risk score medium value. The correlation between clinical variates and the DFS of PRAD was analyzed and calculated using univariate and multivariate Cox with the R package survival v3.1.8. The nomogram was obtained with the R package survival v3.1.8. The C-index analysis was performed with the R package pec v2019.11.3 (21).

### HPA Analysis

Protein levels of six risk model genes expressed in PRAD and normal prostate samples were analyzed using IHC staining data obtained from the HPA database. Four categories of high, medium, low, and not detected were used to evaluate expression levels. These categories included a scoring system based on the proportion of positive-stained cells (>75, 25–75, or <25%) and staining intensity (strong, moderate, weak, or negative).

### Statistical Analysis

All statistical analyses were conducted using R software (version 3.6.1). The Mann–Whitney U-test was used to compare two groups with a non-normal distribution of variables. For comparisons of three groups, Kruskal–Wallis tests of variance were used as nonparametric methods. Correlation analysis was performed using the Person coefficient. All statistical tests were two-sided, and p-values < than 0.05 were considered statistically significant.

## Results

### Metabolism-Associated Subtypes Identified by ssGSEA and PAM Analysis

**Figure 1:**
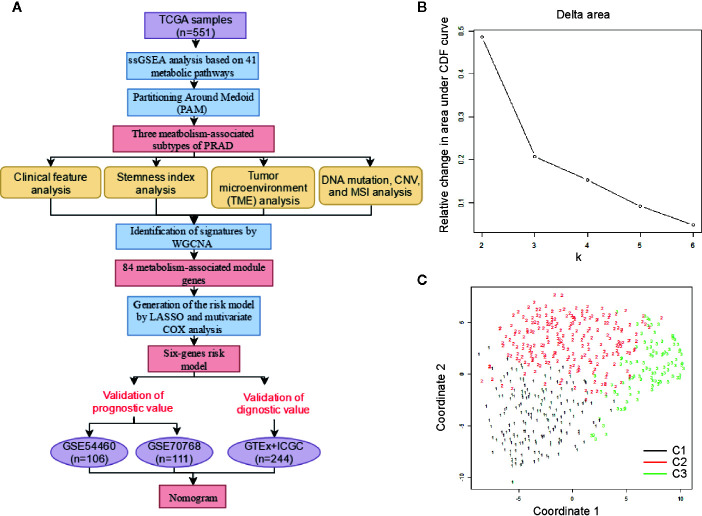
Identification of metabolism-associated subtypes of PRAD using PAM algorithm based on ssGSEA score. **(A)** Workflow in this study. **(B)** Delta area curves for consensus clustering indicating the relative change in area under the cumulative distribution function (CDF) curve for each category number k compared to k − 1. The horizontal axis represents the category number k, and the vertical axis represents the relative change in area under CDF curve. **(C)** t-SNE analysis supported the stratification into three metabolism-associated subtypes of PRAD.

A schematic of our research workflow is shown in **Figure 1A**, and the clinical information regarding the TCGA PRAD cased included in our study is summarized in **Table 1**. To determine the level of activity of the metabolic pathways in each PRAD sample, we calculated the enrichment scores of 41 metabolism-associated gene sets using ssGSEA (**Table S1**). We then performed PAM analysis of ssGSEA scores for 499 PRAD samples and determined the matrix heatmap of the ssGSEA scores retained sharp and clear sides when k = 3, which indicated there were three different metabolism-associated clusters, C1, C2, and C3 (**Figure 1B** and **Figures S1A–F**). To verify the subtype distribution, we performed t-SNE to dimensionally reduce the ssGSEA scores and found the subclass assignment was approximately accordant with the t-SNE coordinates designation (**Figure 1C**).

**Table 1:** Clinical information from the 545 PCa patient of TCGA.

Clinical parameters	Variable	N (total = 545)	Percentages (%)
Age (years)	<=60	242	44.40%
	>60	303	55.60%
T/N grade	T2	188	34.50%
	T3	295	54.13%
	T4	10	1.83%
	unknow (T stage)	52	9.54%
	N0	348	63.85%
	N1	79	14.50%
	unknow (N stage)	118	21.65%
Gleason score	6	48	8.81%
	7	285	52.29%
	8	66	12.11%
	9 & 10	146	26.78%
Survival status	Dead	10	1.83%
	Alive	482	88.44%
	unknown	53	9.72%

**Figure 2:**
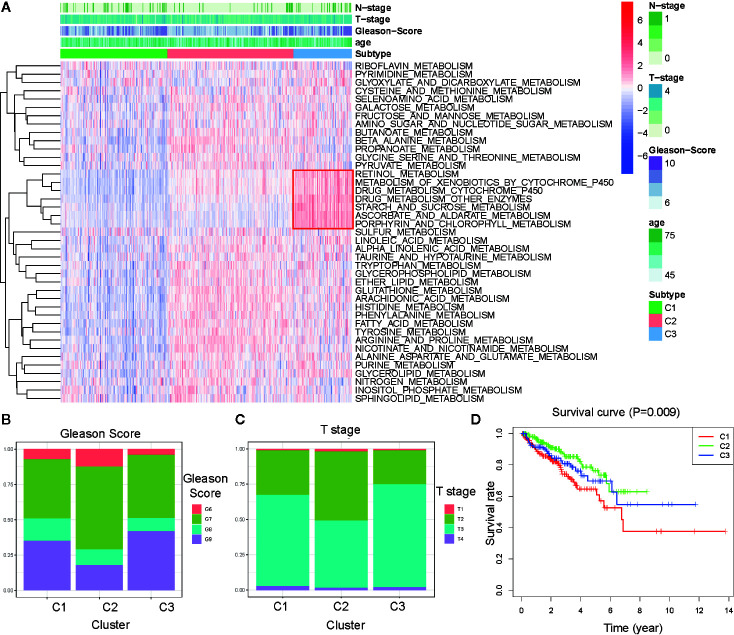
Association between clinical characteristics and the metabolism-associated subtypes. **(A)** Heatmap of the ssGSEA score calculated by metabolic pathways gene sets and specific metabolic pathways of C3 in the red frame. Gleason score **(B)** and Primary Tumor (T) stage **(C)** for each metabolism-associated subtype in the TCGA cohort. The P values are labeled above each boxplot with asterisks (ns represents no significance, ^*^P < 0.05, ^**^P < 0.01, ^***^P < 0.001). **(D)** Survival curves for each metabolism-associated subtype in the TCGA cohort. The horizontal axis represents survival time (year), and the vertical axis represents the probability of survival. The log-rank test was used to assess the statistical significance of the differences between the three subtypes.

To explore the characterization of each subtype, we described the clustering hot map of the metabolic pathway scores (**Figure 2A**). Compared to that of cluster C1, C2 had higher enrichment scores in most metabolic pathways, indicating that tumors from C2 exhibited higher metabolic activity than that of tumors from C1. Concomitantly, the highest specific metabolic pathways scores were observed for cluster C3 and included retinol metabolism, metabolism of xenobiotics by cytochrome P450, drug metabolism cytochrome_P450, drug metabolism other enzymes, starch and sucrose metabolism, ascorbate and aldarate metabolism, and porphyrin and chlorophyll metabolism. The other pathway scores of C3 were higher compared to those of C1, but lower than those of C2. This indicated that tumors from C3 might have had a medium metabolic status at levels between those from C1 and C2 and, at the same time, exhibited some unique metabolic characteristics.

Clinical analysis comparing the different subtypes revealed patients from C1 and C3 had higher primary tumor (T) stage and Gleason scores than patients from C3, but the age and regional lymph nodes (N) stage differences of the patients among these subtypes were not significant (**Figures 2B, C** and **Figures S2A, B**). Prostate-specific antigen (PSA) is the most common index used in the diagnosis and prediction of prognosis for PCa (22). Differences in PSA levels among the three subtypes indicated the subtypes were independent of PSA without any detectable connection (**Figure S2C**). We then performed a K–M survival analysis of patients with PARD from each subtype. The results suggested there were considerable differences in DFS among the three subtypes (p < 0.05; **Figure 2D**). The patients from C1 had the shortest DFS compared to those from the other subtypes. This result indicated that the metabolism-associated subtypes would be associated with different prognoses, and tumors from the different subtypes exhibited considerable differences in their metabolic status.

### Correlation of PRAD Subtypes With Cancer Stem Cell Characteristics

In previous studies, cancer stem cell characteristics have been shown to represent the capability of tumor proliferation and are associated with the development and progression of PRAD (23, 24). To determine the heterogeneity of the current study subtypes, we compared the stemness index of each subtype that was calculated using one-class logistic regression (OCLR) machine learning and ssGSEA. We initially obtained two stemness indices, mRNAsi, and ssGSEAsi (**Table S2**).

**Figure 3:**
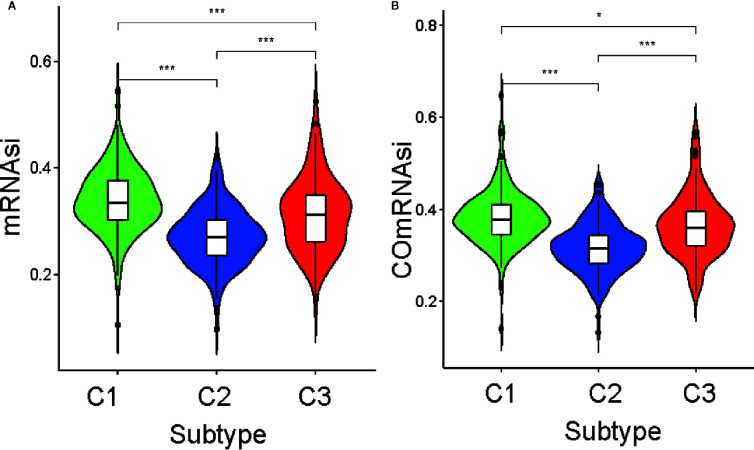
Association between the stemness index and the metabolism-associated subtypes. The pairwise comparison of the mRNAsi **(A)** and COmRNAsi **(B)** between three subtypes. The P values are labeled above each boxplot with asterisks (ns represents no significance, ^*^P < 0.05, ^**^P < 0.01, ^***^P < 0.001).

Differential analysis of mRNAsi indicated there were significant differences among the three subtypes (p <0.05; **Figure 3A**). C1 had the highest stemness index, whereas C2 had the lowest. Moreover, ssGSEAsi analysis indicated that C3 had the highest stemness index (p <0.05; **Figure S3A**). To compensate for the impact of tumor purity on the stemness index, we recalculated the indices using corrected mRNAsi and ssGSEAsi values by dividing them by their respective tumor purity values and then re-performing the differential analysis. The results for the two corrected stemness indices were in approximate accordance with the original results (**Figure 3B** and **Figure S3B**). This suggested the tumors from clusters C1 and C3 had a stronger capacity for invasion, proliferation, and self-renewal compared to that for those from C2.

Because C3 demonstrated the highest ssGSEAsi and COssGSEAsi and had specific metabolic pathways, we performed a correlation analysis between COssGSEAsi/ssGSEAsi and the specific metabolic pathways. The results indicated the starch and sucrose metabolism and porphyrin and chlorophyll metabolism pathways were highly correlated with the PRAD stemness index (cor >0.3; **Figure S3C**).

### Relationship Between PRAD Subtypes and TME

**Figure 4:**
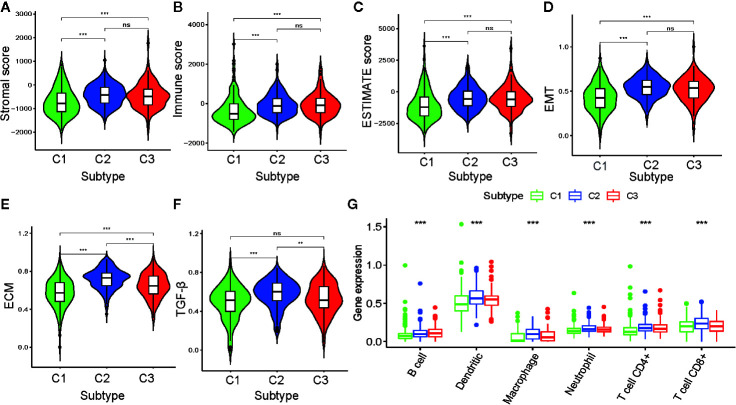
Association between the tumor microenvironment and the metabolism-associated subtypes. The pairwise comparison of the stromal score **(A)**, immune score **(B)**, ESTIMATE score **(C)**, ssGSEA score of EMT **(D)**, ssGSEA score of ECM **(E)**, and ssGSEA score of TGF-β **(F)** between three subtypes. **(G)** The differential analysis of the abundance of immune cells between three subtypes. The P values are labeled above each boxplot with asterisks (ns represents no significance, ^*^P < 0.05, ^**^P < 0.01, ^***^P < 0.001).

To further investigate PRAD tumor heterogeneity, we compared the TME among the metabolism-associated subtypes. In previous studies, TME compounded by both stromal and immune cells played a crucial role in the occurrence and progression of PRAD (25, 26). Moreover, TME may reflect a tumor’s sensitivity to immunotherapies (27, 28). Accordingly, we obtained stromal scores and immune scores for the PRAD tumors in the current study using the ESTIMATE algorithm and then performed a differential analysis of the three subtypes. The results showed that C1 tumors had lower stromal and immune scores compared to those C2 and C3 tumors (**Figures 4A–C**). This suggested that tumor tissue from C1 had higher tumor purity and lower immune infiltration compared to tumor tissues from C2 and C3. To further investigate the differences in stromal cells among the three clusters, we calculated ssGSEA scores for epithelial-mesenchymal transition (EMT), extracellular matrix (ECM), and transforming growth factor-beta (TGF-β) using the corresponding gene sets downloaded from the Molecular Signatures Database (**Table S2**). Differential analysis of these ssGSEA scores suggested C1 tumors had the lowest scores for all three gene sets, which was consistent with the results from the comparison of the stromal scores for the three subtypes (**Figures 4D–F**).

Because of the significant difference in immune scores between PRAD subtypes, we explored immune infiltration to identify their respective immunologic landscapes. The abundance of six immune-related cell types, B cell, dendritic, macrophage, neutrophil, CD4+ T cell, and CD8+ T cell, was download from TIMER2.0. Significant differences for all six immune cell types were verified among the cluster subtypes, with the tumors from C2 having the highest abundance of all immune cells, except B cells. The cluster with the highest abundance of B cells was C3, whereas C1 tumors had the lowest abundance of all the immune cell types evaluated (**Figure 4G**). Our results indicated that the metabolism-associated subtypes of PRAD exhibited remarkably distinct characteristics with respect to immune infiltration.

### PRAD Subtype Relationship With DNA Mutations, CNV, and MSI

**Figure 5:**
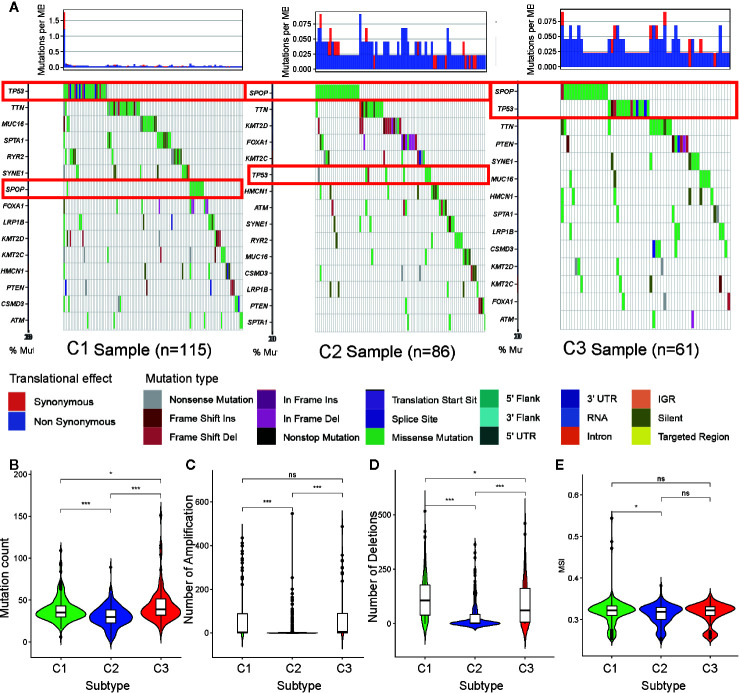
Association between metabolism-associated subtypes and DNA mutation and copy number variation. **(A)** The waterfall plot of the top 15 genes of DNA mutation in three subtypes. The pairwise comparison of the mutation count **(B)**, the number of amplification **(C)**, the number of deletions **(D)**, and MSI **(E)** between three subtypes. The P values are labeled above each boxplot with asterisks (ns represents no significance, ^*^P < 0.05, ^**^P < 0.01, ^***^P < 0.001).

To determine the reason for cluster subtype heterogeneity, we investigated whether differences existed among the three subtypes in DNA mutation burdens and patterns of somatic mutation rates. By displaying the 15 genes determined to have the highest frequency of DNA mutations in PRAD in a waterfall plot, we observed remarkably different landscapes for each of the PRAD subtype (**Figure 5A**). Mutation of *TP53* was the most frequent DNA mutation in cluster C1 and mutation of *SPOP* was the most frequent in cluster C2. These results indicated that the *TP53* mutation was a characteristic mutation of C1 tumors, and the *SPOP* mutation was a characteristic mutation of C2 tumors. The C3 cluster had high mutation rates of both *SPOP* and *TP53*. This may explain why tumors from C3 exhibited a status between those of C1 and C2, regardless of the stemness index or TME analysis.

Tumor mutation burden (TMB) is considered to reflect the sensitivity of tumors to targeted drug therapies (29). To further investigate the features of DNA mutations and clinical treatment options for PARD subtypes, we compared the differences in the number of DNA mutations among the subtypes. We found that tumors of subtypes C1 and C3 had higher mutation counts than those of subtype C2 (**Figure 5B**). This further indicated that tumors from C1 and C3 exhibited greater heterogeneity compared to those from C2.

CNV occurring upstream of genes regulates gene expression and influence tumor occurrence and development (30). To further explore whether this DNA element may lead to increased heterogeneity among the metabolism-associated subtypes, we downloaded a list of metabolism-relevant genes (31) and analyzed the number of amplifications and deletions regarding the CNV in these genes. We found that the number of CNV amplifications and deletions was highest in subset C1, followed by that in subset C3, with the fewest being observed in subset C2 (**Figures 5C, D**). These results suggest that CNV results in significant heterogeneity among the three subtypes.

Previous studies have shown that MSI is a crucial indicator of genome instability and is associated with many genetic diseases (32). In our studies, we obtained level data from MSI of each PRAD sample calculated in a previous study (33) and performed differential analysis. The results indicate that C1 has a higher level of MSI than C2 (p <0.05) (**Figure 5E**), suggesting that MSI may be the resource of tumor heterogeneity in C1.

### Identification of Metabolism-Associated Signatures

**Figure 6:**
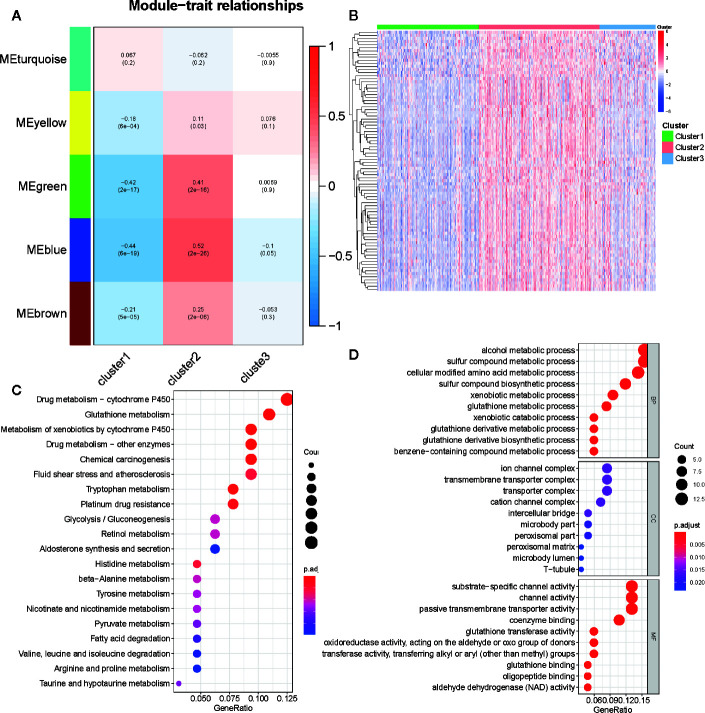
Identification of metabolism-associated module genes of PRAD in the WGCNA and the functional enrichment analysis of these genes. **(A)** Heatmap of the correlation between module Eigengenes and metabolism-associated subtypes (C1, C2, and C3). **(B)** Heatmap of 84 metabolism-associated module genes in three subtypes. **(C)** The GO analysis of metabolism-associated module genes. **(D)** The KEGG analysis of metabolism-associated module genes.

We selected 2,029 metabolism-associated genes among the TCGA PRAD cohort samples and constructed a co-expression network through co-expression analysis (31). Average linkage hierarchical clustering identified five modules. To realize the scale-free co-expression network, a power of β = 3 was used (**Figure S4A**). We then adopted the dynamic hybrid tree cut method to combine highly similar modules using a cutoff value = 0.25 and module size = 50 (**Figure S4B**). Although we failed to identify a module associated with the C3 subtype, the green and blue modules showed a strong association with C1 and C2 subtypes (cor > 0.3 or <−0.3; **Figures 6A** and **S4C**). Ultimately, 489 associated genes were identified, including 388 genes in the blue module and 101 genes in the green module. Of the 489 genes, 84 (cor of GS with C1 and C2 >0.3 or <−0.3) were determined as metabolism-associated module genes (**Figure 6B** and **Table S3**).

To determine the biochemical functions of the metabolism-relevant module genes, we performed GO and KEGG function enrichment analyses. Some metabolic pathways, such as alcohol metabolic process, sulfur compound metabolic process, cellular modified amino acid metabolic process, phenylalanine metabolism, drug metabolism cytochrome P450, and glutathione metabolism were significantly enriched by these genes (**Figures 6C, D**; **Table S4**). This further confirmed that the functions of the selected genes were closely associated with PRAD metabolism.

### Metabolism-Associated Risk Model Development and Validation

Patients with DFS <20 d and those without available DFS information were excluded. A total of 489 patients in the TCGA database were included in the training cohort of this study (**Table 2**). We performed a univariate Cox regression analysis of the 84 previously selected variables to identify potential optimal prognostic targets. A total of 23 genes that met the prognostic criteria were identified (p <0.05; **Table S5**). To avoid overfitting of the model. The prognostic biomarkers that highly correlated with one another were first removed using LASSO regression, resulting in six candidate prognostic genes (**Figures S5A, B**). These six genes were then analyzed using the multivariate Cox proportional hazards regression method. Finally, metabolism-associated module genes related to DFS of PRAD were identified, including *CACNG4*, *SLC2A4*, *EPHX2*, *CA14*, *NUDT7*, and *ADH5* (**Table 3**). The formula used for calculating the risk score was as follows:

$$\begin{array}{l} \text{Risk}\;\text{score}=(-0.0.83\times \text{FPKM}\;\text{of}\;CACNG4)+(-0.0980\times \text{FPKM}\;\text{of}\;SLC2A4)\\ +(-0.0161\times \text{FPKM}\;\text{of}\;EPHX2)+(-0.2182\times \text{FPKM}\;\text{of}\;CA14)+(-0.2055\times \text{FPKM}\;\text{of}NUDT7)\\ +(-0.0213\times \text{FPKM}\;\text{of}\;ADH5) \end{array}$$

**Table 2:** Grouping of PCa patients for survival analysis.

Clinical parameter	Variable	TCGA	GSE 54460	GSE 70768
Recurrence or no	Recurrence	91 (18.61%)	51 (48.11%)	19 (17.11%)
	No recurrence	398 (81.39%)	55 (51.89%)	92 (82.88%)

**Table 3:** Risk genes in the prognostic risk model.

GENE	Coef	HR	HR.95L	HR.95H	P value
CACNG4	−0.00837	0.991664	0.96811	1.015792	0.49494
SLC2A4	−0.09798	0.906668	0.768444	1.069754	0.245648
EPHX2	−0.01612	0.984005	0.960761	1.007812	0.18618
CA14	−0.21817	0.803992	0.352487	1.833836	0.604063
NUDT7	−0.20554	0.814211	0.501774	1.321193	0.405298
ADH5	−0.02131	0.978912	0.95889	0.999352	0.043232

**Figure 7:**
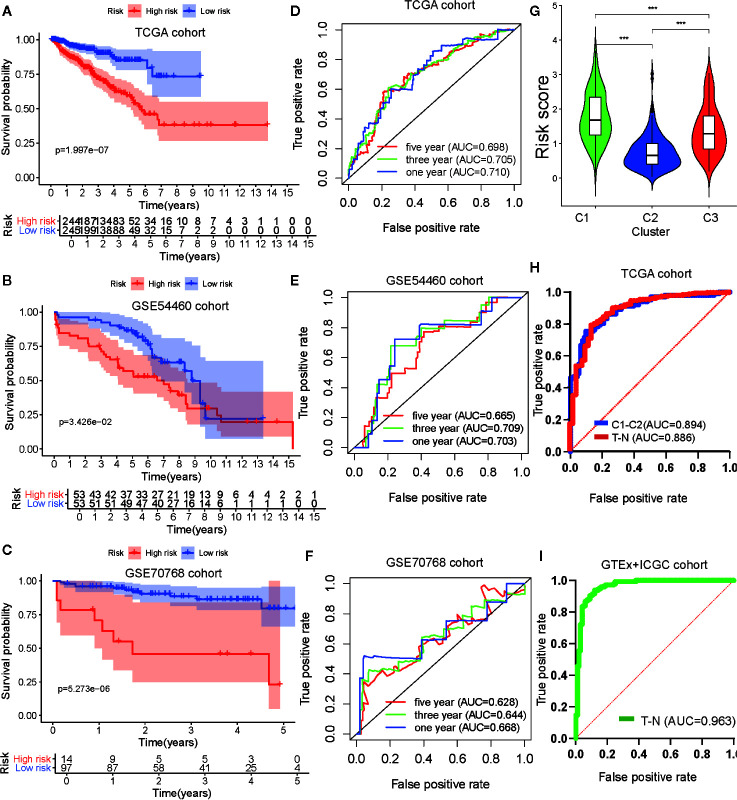
Development and validation of the metabolism-associated risk model. Kaplan–Meier curve analysis of high-risk the low-risk groups in the TCGA cohort **(A)**, in the GSE54460 cohort **(B)**, and in the GSE70768 cohort **(C)**. Time-dependent ROC curve analysis of the prognostic model in the TCGA cohort **(D)**, in the GSE54460 cohort **(E)**, and in the GSE70768 cohort **(F)**. **(G)** The differential analysis of risk score between three subtypes in PRAD. The P values are labeled above each boxplot with asterisks (ns represents no significance, ^*^P < 0.05, ^**^P < 0.01, ^***^P < 0.001). **(H)** ROC curve analysis of risk score to predict sample types (tumor and normal tissue) and metabolism-associated subtypes (C1 and C2) in TCGA. **(I)** ROC curve analysis of risk score to predict sample types (tumor and normal tissue) in GTEx and ICGC cohorts.

To verify the robustness of the risk model, two external cohorts available in the GEO repository, datasets GSE54460 and GSE70768, were obtained and used as validation cohorts. Each cohort was separated into two groups according to the median value of each risk score. To evaluate the differences in prognosis between the high-risk and low-risk groups, a K–M survival curve was constructed based on the log-rank test. Patients in the high-risk group of the TCGA cohort GSE54460 dataset exhibited poorer outcomes compared to those in the low-risk group (p < 0.05; **Figures 7A, B**). As for the GSE70768 cohort, the arrangement characteristic of microarray data differed from RNA-seq data. Therefore, we divided the cohort according to most cutoff value, which was calculated using X-tile, and found there was a significantly different prognosis between the high-risk and low-risk groups (**Figure 7C**). We used a time-dependent ROC curve to investigate the predictive accuracy of our model and determined the AUC of the prognostic model using the TCGA training cohort was 0.769 at one year, 0.702 at three years, and 0.705 at five years (**Figure 7D**). For the testing cohorts, the AUC of the prognostic model for the GSE54460 cohort was 0.703 at one year, 0.709 at three years, and 0.665 at five years and 0.668 at one year, 0.644 at three years, and 0.628 at five years for the GSE70768 cohort (**Figures 7E, F**).

### Identification of Risk Model Biomarkers Biological Functions and Construction of Nomogram

GSEA analysis was performed to explore the biological functions of the risk model genes. The results indicated the genes had a significant relationship with cell cycle, DNA replication, homologous recombination, RNA degradation, and spliceosome pathways in the high-risk group and with beta alanine metabolism, dilated cardiomyopathy, drug metabolism cytochrome P450, metabolism of xenobiotics by cytochrome_P450, and vascular smooth muscle contraction pathways in the low-risk group (**Figures S5C, D**; **Table S6**). This suggested our risk model genes influenced these pathways and that these pathways may impact PRAD DFS.

We then explored the relationship between clinical/metabolism-associated subtypes and risk scores and found there was a close relationship between risk score and age/Gleason score/T stage/N stage/metabolism-associated subtypes (**Figures S5E–H** and **Figure 7G**). This suggested that our risk model had predictive value, not only in PRAD DFS, but also in tumor size, lymphatic node metastasis, and metabolism-associated subtype. Meanwhile, when we used ROC to determine whether our risk model could predict sample type (tumor vs. normal tissue) and metabolism-associated subtypes (C1 vs. C2), we observed that the AUC values of the risk model were 0.886 and 0.894, respectively, in TCGA cohort (**Figure 7H**). To determine the diagnostic value of our model, we used ROC to evaluate samples from the external group (GTEx and ICGC) and found that there was an outstanding predictor value for PCa diagnosis with our model (AUC = 0.963) (**Figure 7I** and **Table 4**).

**Table 4:** Grouping of PCa patients for diagnostic analysis.

Clinical parameter	Variable	TCGA	GTEx + ICGC
Normal or tumor tissue	Normal	52 (10.60%)	100 (40.98%)
	Tumor	499 (89.40%)	144 (59.02%)

Finally, we selected clinical variates with independent prognostic value to obtain a nomogram through univariate and multivariate Cox analyses (**Figure S5I** and **Table S7**). ROC analysis and C-index calculation assessed the clinical meaning of the nomogram and suggested that the clinical nomogram had a better net benefit than clinical variate or risk score only models (**Figures S5J, K**).

**Figure 8:**
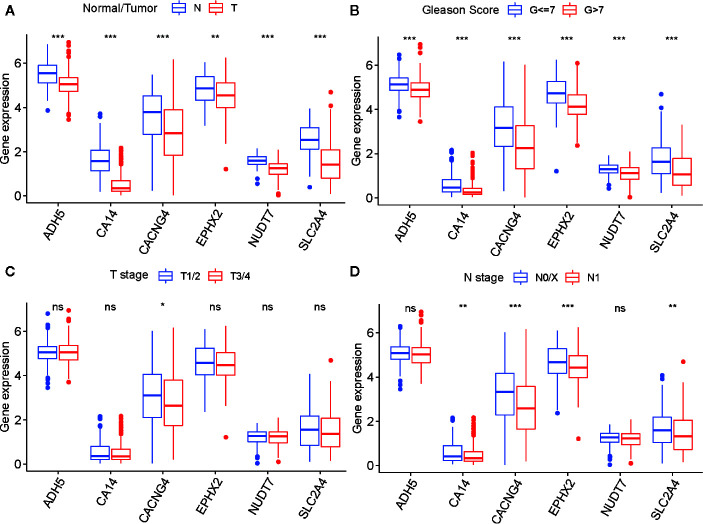
The differential analysis of six risk model genes between PRAD tissue and normal prostate tissue **(A)**, different Gleason score PRAD samples **(B)**, different Primary Tumor (T) stage samples **(C)**, and different Lymph Nodes (N) stage samples **(D)** in TCGA cohort. The P values are labeled above each boxplot with asterisks (ns represents no significance, ^*^P < 0.05, ^**^P < 0.01, ^***^P < 0.001).

To determine the diagnostic and prognostic value of a single risk model gene, we performed differential analyses of six risk model genes between different types, Gleason score, T- and N stage of samples, and K–M analyses of each risk model gene in the TCGA cohort. The findings indicated that six risk model genes have differing expression levels between normal and tumor tissue, and different Gleason score samples. Simultaneously, there was a significant difference in prognosis between PARD patients with high-risk and low-risk model gene expression (**Figures 8A–D** and **Figures S6A–F**).

To confirm whether there was a differential abundance of proteins associated with the selected genes between normal prostate tissues and tumor tissues of patients with PRAD, we downloaded IHC micrographs from the HPA database. Three of the six risk model genes were found to exhibit differential staining between normal prostate tissue and PRAD tissue, those being *EPHX2*, *NUDT7*, and *ADH5* (**Figures S7A–C**). The results suggested that the expression of these proteins was decreased in PRAD tissues. This was in accordance with the differential analysis of expression for the six risk model genes of the TCGA cohort and further indicated these genes might play crucial roles in the occurrence and development of PRAD.

### Drug Sensibility Analysis With Metabolism-Associated Subtypes and Risk Model

**Figure 9:**
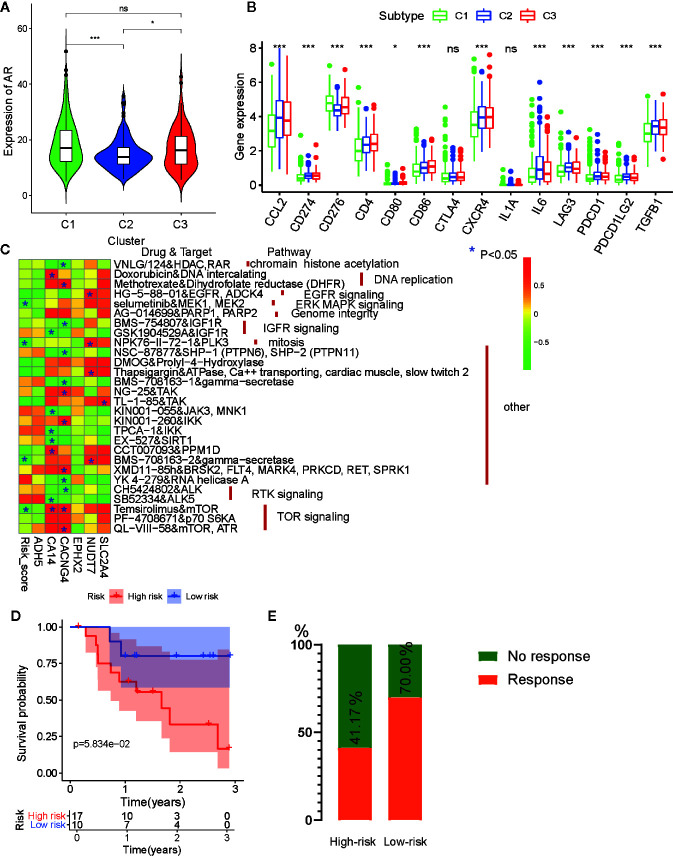
Drug sensibility analysis with metabolism-associated subtypes and risk model. **(A)** The pairwise comparison of the androgen receptor (AR) expression between three subtypes. **(B)** The differential analysis of the expression of 14 checkpoints between three subtypes. The P values are labeled above each boxplot with asterisks (ns represents no significance, ^*^P < 0.05, ^**^P < 0.01, ^***^P < 0.001). **(C)** Heatmap for correlation between drug sensitivity and expression levels of six risk model genes. ^*^p < 0.05. **(D)** Survival analyses for low-risk and high-risk scores patient groups in the anti-PD1 immunotherapy cohort using Kaplan–Meier curves. **(E)** The proportion of patients with response to PD-1 blockade immunotherapy in low-risk or high-risk scores groups.

Anti-androgen treatment is the first non-surgical treatment for PRAD (34). It has been shown that the level of the androgen receptor (AR) gene expression in tumor tissue is closely related to anti-androgen treatment sensitivity (35). Therefore, we also compared the expression levels of AR in the three subtypes and found that tumors from C1 and C3 had higher AR expression levels than those from C2 (**Figure 9A**). This indicates that patients from C1 and C3 may be more sensitive to anti-androgen treatment that those from C2, and that there existed considerable tumor heterogeneity among the subtypes.

For castration-resistant prostate cancer, immunotherapy and chemotherapy are the preferred treatments. To explore the sensitivity of each cluster subtype to immunotherapies, we collected data for 14 immune checkpoints and compared the gene expression levels of these proteins. Significant differences in gene expression of the checkpoint proteins among the three subtypes were observed, except for CTLA4 and IL1A. Cluster C1 exhibited lower expression of most the checkpoint genes relative to that cluster C2 and C3 (**Figure 9B**), which is in accordance with the immune infiltration status described above. Therefore, we were able to determine that tumors of the C1 subtype demonstrated lower immune infiltration and may, therefore, garner fewer benefits from treatment with immunotherapies.

Then, to evaluate the association between drug sensitivity and the metabolism-associated risk model, correlation analysis was performed using half-inhibitory concentration (IC50) of chemotherapeutic drugs, risk score, and gene expression data from six PCa cell lines obtained from the GDSC database. As a result, four anti-tumor drugs (selumetinib, NPK76-11-72-1, BMS-708163, and temsirolimus) were negatively correlated with risk score (**Figure 9C** and **Table S8**). Among the six genes, CA14, CACNG4, and NUDT7 had associations with more than four drugs, indicating that these genes and risk scores may guide chemotherapy in drug choice.

Finally, immunotherapies represented by PD-L1 and PD-1 blockades have undoubtedly emerged as a breakthrough in cancer therapy (36, 37). We explored whether our risk model could predict patient responses to immune checkpoint blockade therapy based on the anti-PD-1 cohort (GSE78220). First, we performed K-M survival analysis and found that melanoma patients with high-risk scores had shorter OS than patients with low-risk scores, although no significant difference was observed (p = 0.058) (**Figure 9D**). The proportion of beneficiaries with anti-PD-1 treatment in the high-risk cohort (41.17%) was lower than that of the low-risk cohort (70%) (**Figure 9E**). These results implied that patients with low-risk scores would get more benefits from immunotherapy than patients with high-risk scores.

## Discussion

With the development of RNA-seq technology, many classical analyses based on gene expression data have been reported for most cancers (6, 38–40). However, few cluster studies have been performed on PCa to explore the tumor metabolic characteristics. Thus, we identified in our current work a metabolism-associated PRAD classification based on ssGSEA and 41 metabolic pathway gene sets. The PRAD cases included in the study were divided into three subtypes. The metabolic features, clinical characteristics, prognosis, TME, stemness index, DNA mutation, CNV, and MSI were then investigated in the different subtypes. Subtype C1 exhibited low metabolic levels and was similar to high-grade PCa with high tumor purity and low immune infiltration. Furthermore, patients from C1 had worst prognosis and the shortest DFS among the patients with PCa. In comparison, patients from C2 displayed high metabolic levels in most pathways and were similar to low-grade PCa with low tumor purity, low stemness index, and high immune cell infiltration. Patients with tumors from C2 had the best prognosis and longest DFS among the patients with PCa. Patients from C3 represented a medium state between the findings for those from C1 and C2 and demonstrated similar medium-grade PCa. Specifically, C3 had highly metabolic pathways activity and the highest stemness index. Accordingly, we believe there may be some connections between starch/sucrose/porphyrin/chlorophyll metabolism and a high stemness index.

Cancer stem cells play important roles in therapeutic responses and the progression of cancer (41). To further explore the reason C1 subset has the worst prognosis, we continued investigating the stem index between three subtypes. C1 and C3 had higher stemness indices, which indicates more malignant tumors from these subsets compared to those from C2. This may partly explain why patients with PCa from subsets C1 and C3 presented with shorter DFS and worse prognosis compared to that of patients from subset C2. These results indicate a remarkable tumor heterogeneity among PRAD metabolism-associated subtypes. However, the reason for C3 tumors having the highest ssGSEAsi is unclear. In previous studies, starch and sucrose metabolism is associated with the progression of colon cancer (42). Therefore, we believe that specific metabolic pathways, such as those for starch/sucrose/porphyrin/chlorophyll metabolism, have crucial roles in tumor progression. This hypothesis needs to be tested using *in vitro* experiments and single-cell sequencing.

TME is a remarkable factor impacting the occurrence and development of PCa. Many cancer-promoting factors play a role in the EMT pathway (43). For instance, the expression of PDL1 can affect the prognosis of adrenocortical carcinoma (44). Regardless of stromal or immune cells, the C2 and C3 subtypes displayed more characteristics of TME. Stromal scores indicated that C2 and C3 had greater stromal cell content than C1. ECM functions as cell scaffoldings and can induce EMT in stromal cells, with the TGF-β pathway having a strong connection with this process (45). To further explore the features of stromal cells in PCa, ECM, EMT, and TGF-β, ssGSEA scores were calculated for each gene set. Differential analysis suggests that C2 had the most significant activity in this process. EMT often has a close relationship with cell cancer and poor prognosis (46–48); however, C2 had the best prognosis among the three subtypes. Therefore, we believe that EMT does not have an obvious cancer-promoting function in PCa. As for the increase of EMT in C2, we believe this phenomenon was the result of increased stromal cell content in C2. The immune system is the most important anti-cancer system in the body (49). In a previous study, immune cells were found to be strongly lethal in fighting tumor cells. Natural killer cells can kill lung cancer tumor cells and are regulated by TME (50). Additional support that the immune system is important is demonstrated by T cells being able to be used in clinical settings for the treatment of cancer (51). Herein, our study shows that the levels of all immune cells (B cells, dendritic cells, macrophages, neutrophils, CD4+ T cells, and CD8+ T cells) were increased in C2, indicating that tumors from C2 were in a state of immune activation. We believe this is the reason C2 had the best prognosis among the three subtypes.

TMB is presumed to have a close relationship with tumor heterogeneity (52). *TP53* is the most prominent gene in pan-cancer investigations. For instance, *TP53* mutations lead to high-grade cancer and tumor heterogeneity of ovarian granulosa (53). At the same time, mutations in *TP53* were shown to be strongly associated with the occurrence and progression of PCa (54). *SPOP* mutations have also been considered for their impact on castration sensitivity in PCa (55). Gene mutation spectra were significantly different among the three metabolism-associated subtypes in our current study. For instance, in C1, the *TP53* mutation rate was higher than that of others, whereas C2 had a high mutation rate of *SPOP*. This indicates that the tumor features of C1 and C2 partly result from *TP53* and *SPOP* mutations, respectively. C3 had high mutation rates of both *TP53* and *SPOP*, which further supported that C3 exhibited an intermediate state between C1 and C2. Meanwhile, many studies have investigated CNV in PCa, with the results indicating that CNV can affect tumor features and heterogeneity (56). MSI also has been regarded as a vital factor in DNA mismatches and can improve tumor heterogeneity in many types of cancer. Herein, we show that there is a significant difference in CNV of metabolic genes and MSI levels among the three subtypes. Our current work revealed that there was a significant difference in CNV of metabolic genes among the three subtypes. C1 had the highest number of amplifications and deletions. This indicates that the *TP53* mutation, *SPOP* mutation, and increased CNV and MSI were key factors contributing to the tumor heterogeneity observed among the subtypes.

A general opinion regarding tumor cells with high activity levels of one or more specific metabolic pathways is that they have a stronger capacity for invasion, proliferation, and self-renewal compared to cells with low metabolic activity. For instance, higher sulfur amino acid metabolic levels in liver cancer can accelerate the EMT process and cancer cell migration (57). Aldehyde oxidase 1 decreases the metabolic level and displays tumor inhibition activity in bladder cancer (58), whereas long intergenic non-coding RNA-nucleotide metabolism regulator upregulates nucleotide metabolism and increase the proliferation of tumor cells (59). Our results partly contradict these views in that we found tumors from C1 with the lowest metabolic activity level had the worst prognosis in patients with PCa. The samples from patients with PCa were mixed tissues, including tumor cells, normal prostate cells, stromal cells, and immune cells. Tumor malignancy was determined based on several factors, including tumor purity, tumor proliferation ability, and the TME *in vivo*. Thus, our results differed in part from those concluded *in vitro*, where tumor malignancy was determined by the tumor cells only. Normal prostate cells are smooth muscle cells that exhibit high metabolic levels, whereas tumor cells may show lower levels of metabolic activity compared to normal prostate cells. Meanwhile, ssGSEA scores based on mRNA-seq data from second-generation sequencing reflects the metabolic level of the whole sample, rather than only tumor cells. Therefore, in this study, we determined a lower metabolic level, and that greater tumor heterogeneity of the PCa samples was associated with a worse prognosis for the patient. This was in accordance with that reported for liver cancer (60). These specifications may partly explain why tumors from the C1 subset had the worst prognosis in PCa.

According to the above subtype analyses, we consider that C2 is the subtype that characterizes early PCa. In fact, during the early stage, the tumor metabolic status often resembles that of normal tissue. Besides, during the initial stage, the immune system exerts a strong anti-tumor response, and the tumor has low heterogeneity and stemness index. On the contrary, due to immune escape and the decrease of blood supply in the later stage of tumor progression, immune cells are not able to infiltrate the tumor. Thus, C1 has milder immune characteristics than C2. Nevertheless, because C1 is an advanced tumor, it had the highest tumor heterogeneity and the lowest metabolic status among the three subtypes. As for C3, which is regarded as an intermediate between C1 and C2, we consider it to be the crucial status of PCa from early to advanced tumor stage. The unique metabolic pathways of C3 reportedly affect the malignant transformation of healthy tissue.

To predict prognosis, previous studies have developed prognostic risk models for PCa based on gene expression data (61–63). This indicates that risk models based on high-throughput data may accurately predict the prognosis of PCa. Therefore, we used WGCNA to identify the characteristic genes of C1 and C2. Using multiple algorithms, a six-gene risk model, including *CACNG4*, *SLC2A4*, *EPHX2*, *CA14*, *NUDT7*, and *ADH5*, was established using the TCGA cohort. Through the testing of four external datasets, our metabolism-associated risk model was demonstrated to have strong robustness. GSEA analysis provided further evidence that our risk genes are related to PCa metabolism. Finally, we combined risk score and clinical variates to obtain a nomogram to help clinicians predict the DFS for PCa patients.

Many studies have demonstrated that gene expression data can be used to predict drug treatment sensitivity. For instance, molecular profiling can be used to identify treatment-refractory metastatic castration-resistant prostate cancer (64). C1 had the highest AR expression. This indicates that C1 may exhibit high sensitivity to anti-androgenic therapy. With a high expression of immune checkpoints and significant features of immune cell infiltration, C2 tumors may benefit more from immune-targeted therapy, whereas C3, with an active status of specific drug metabolism pathways, may facilitate the development of tolerance to traditional chemotherapy (65). In the drug sensitivity analysis of the risk model, our study suggests that patients with low-risk scores may benefit more from anti-PD-1 treatment, and this is consistent with C2 being more suitable for immune target therapy. All of these conclusions need to be validated *in vitro*.

In contrast to previous research that focused on the metabolic level of single tumor cell types, we explored the metabolic features of mixed cancer samples. We first investigated the characteristics of metabolic pathways using cluster analysis and explored tumor heterogeneity in multiple dimensions employing multi-omics. Finally, our risk model of PCa was constructed and verified using a large number of samples and multiple datasets. However, our research also had limitations. First, the data we studied were from public databases rather than our database. Second, we did not perform *in vitro* or *in vivo* experiments to further investigate the mechanism of metabolism-associated genes in PCa. These are what we plan to do next.

## Conclusions

Three metabolism-associated subtypes were first identified by unsupervised cluster and ssGSEA analyses in PCa. Differential analyses indicated these subtypes could reflect tumor heterogeneity in the stemness index, tumor microenvironment, TMB, CNV, MSI, and clinical features. So our metabolism-associated subtypes can better represent the metabolic characteristics of PCa and can be beneficial in exploring the metabolic mechanism of occurrence and development of PCa. Meanwhile, a six-gene metabolism-associated risk score model by using four separate datasets and demonstrated strong robustness in the prediction of sample types (tumor and normal tissue), DFS, metabolism-associated subtypes, and anti-tumor therapeutic effect. Therefore our model can powerfully help clinicians evaluate the prognosis and develop personalized treatment for PCa patients. Although the six prognostic markers still require experimental verification, they may provide insight and a prospect for further investigation and clinical work regarding PCa.

## Data Availability Statement

Publicly available datasets were analyzed in this study. This data can be found here: https://portal.gdc.cancer.gov/ (PRAD); https://www.ncbi.nlm.nih.gov/gds/ (GSE54460, 70768, 78220); https://www.cancerrxgene.org/ (22RV1, DU-145, LNCaP-Clone-FGC, PC-3, PWR-1E, and VCaP); https://www.gtexportal.org/ (Prostate); https://icgc.org/(PRAD).

## Author Contributions

YZ designed the study and analyzed the data. YZ and RZ revised the images. YZ, RZ, and FL performed the literature search and collected data for the manuscript. XL and LZ revised the manuscript. All authors contributed to the article and approved the submitted version.

## Funding

This work was supported by the National Natural Science Foundation of China (No. 81771768).

## Conflict of Interest

The authors declare that the research was conducted in the absence of any commercial or financial relationships that could be construed as a potential conflict of interest.

## Supplementary Material

Supplementary Figure 1 The PAM analysis of ssGSEA score based on metabolism-associated pathways. **(A)** Consensus among clusters for each category number K. Color-coded heatmap corresponding to the consensus matrix for k = 2 **(B)**, K = 3 **(C)**, K = 4 **(D)**, K = 5 **(E)**, and K = 6 **(F)** obtained by applying consensus clustering.

Supplementary Figure 2 Association between clinical characteristics and the metabolism-associated subtypes. Age **(A)** and Lymph Nodes (N) stage **(B)** for each metabolism-associated subtype in the TCGA cohort. **(C)** The pairwise comparison of the PSA between three subtypes. The P values are labeled above each boxplot with asterisks (ns represents no significance, *P <0.05, **P <0.01, ***P <0.001).

Supplementary Figure 3 Association between the stemness index and the metabolism-associated subtypes. The pairwise comparison of the ssGSEAsi **(A)** and COssGSEAsi **(B)** between three subtypes. The P values are labeled above each boxplot with asterisks (ns represents no significance, *P <0.05, **P <0.01, ***P <0.001). **(C)** The correlation analysis between specific metabolic pathways of C3 and ssGSEAsi in the TCGA cohort.

Supplementary Figure 4 The WCGNA analysis of metabolism-associated genes among three subtypes. **(A)** Analysis of the scale-free fit index and the mean connectivity for various soft-thresholding powers, and checking the scale-free topology when β = 3. K represents the logarithm of whole network connectivity, p(k) represents the logarithm of the corresponding frequency distribution. K is negatively correlated with p(k) (correlation coefficient = 0.88), which represents scale-free topology. **(B)** Identification of a co-expression module in PCa. The branches of the cluster dendrogram correspond to the five different gene modules. Each piece of the leaves on the cluster dendrogram corresponds to a gene. **(C)** Scatter plot of module eigengenes in the blue and green modules.

Supplementary Figure 5 Identification of risk model functions and development of the nomogram. **(A**, **B)** prognostic genes selected through Lasso regression. **(C, D)** Enrichment plots of the top five KEGG pathways in the high-risk score and low-risk score groups in PRAD. The relationship between the age **(E)**/Gleason score **(F)**/N stage **(G)**/T stage **(H)** and risk score in the TCGA cohort. **(I)** Nomogram for predicting the probability of 1, 3, and 5 disease-free survival times for PCa patients. **(J)** ROC analysis for nomogram in 1, 3, and 5 years. **(K)** The C-index analysis for clinical variates, risk model, and nomogram.

Supplementary Figure 6 The K-M survival analysis of six risk model genes. **(A)** ADH5. **(B)** CA14. **(C)** CACNG4. **(D)** EPHX2. **(E)** NUDT7. **(F)** SLC2A4.

Supplementary Figure 7 The protein expression of three risk model genes in normal prostate tissues and PRAD tissues from the Human Protein Atlas (HPA) database. **(A)** ADAH5. **(B)** EPHX2T. **(C)** NUDT7N.

Supplementary Table 1 The 41 metabolism pathway gene sets collected to evaluate the metabolic status of PRAD samples.

Supplementary Table 2 The ssGSEA score of each sample of PRAD in TCGA based on metabolic pathways, stem cell, EMT, ECM, and TGF-β gene sets.

Supplementary Table 3 The WGCNA analysis between the expression of metabolism-associated genes and metabolism-associated subtypes in PRAD.

Supplementary Table 4 The GO and KEGG functional enrichment analysis of metabolism-associated module genes.

Supplementary Table 5 The Univariable Cox analysis of metabolism-associated prognostic genes.

Supplementary Table 6 The GSEA analysis of risk score in TCGA.

Supplementary Table 7 The Univariable and Multivariable Cox analysis of clinical variates and risk score.

Supplementary Table 8 Drug sensitivity analysis of risk model genes and risk score.

## Abbreviations

PCa, prostate cancer; DFS, disease-free survival; TCGA, The Cancer Genome Atlas; PRAD, prostate adenocarcinoma; GTEx, Genotype-Tissue Expression; ICGC, International Cancer Genome Consortium; GEO, Gene Expression Omnibus; ssGSEA, single-sample gene set enrichment analysis; PAM, partitioning around medoid; t-SNE: t-distributed stochastic neighbor embedding; GSEA, gene set enrichment analysis; K–M, Kaplan–Meier survival analysis; WGCNA, weighted gene co-expression network analysis; LASSO, least absolute shrinkage and selection operator; TME, tumor microenvironment; MSI, microsatellite instability; FPKM, fragments per kilobase of transcript per million fragments; RNA-seq, RNA sequencing; GDSC, Genomics of Drug Sensitivity in Cancer; IHC, immunohistochemistry; HPA, Human Protein Atlas; MSigDB, Molecular Signatures Database; TIMER2.0, Tumor IMmune Estimation Resource version 2.0; KEGG, Kyoto Encyclopedia of Genes and Genomes; t-SNE, t-distributed stochastic neighbor embedding; mRNAsi, mRNA expression-based stemness index; ssGSEAsi, ssGSEA stemness index; AM, adjacency matrix; TOM, topological overlap matrix; MEs; module eigengenes; GS, gene significance; GO, gene ontology; ROC, receiver operating characteristic; AUC, area under the ROC curve; PSA, prostate-specific antigen; AR, androgen receptor; TMB, tumor mutation burden; AR, androgen receptor; RP, radical prostatectomy.
