# Supplementary figures and images for: Identification of Metabolism-Associated Prostate Cancer Subtypes and Construction of a Prognostic Risk Model
Source: Front Oncol. 2020 Nov 26;10:598801. doi: 10.3389/fonc.2020.598801 (PMC7726320; doi:10.3389/fonc.2020.598801)

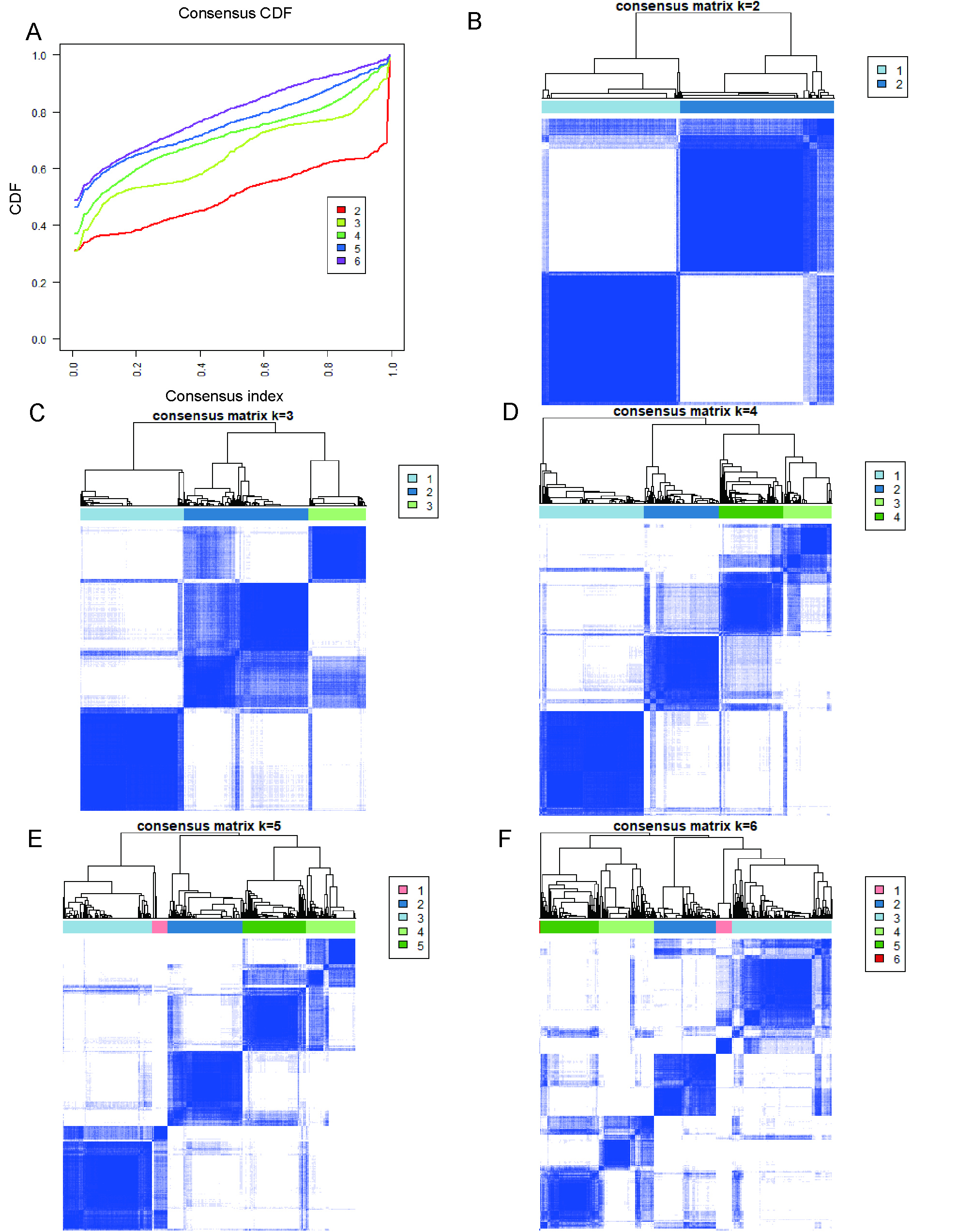

Supplement: Supplementary Figure 1 — The PAM analysis of ssGSEA score based on metabolism-associated pathways. (A) Consensus among clusters for each category number K. Color-coded heatmap corresponding to the consensus matrix for k = 2 (B), K = 3 (C), K = 4 (D), K = 5 (E), and K = 6 (F) obtained by applying consensus clustering. [file Image_1.tif]

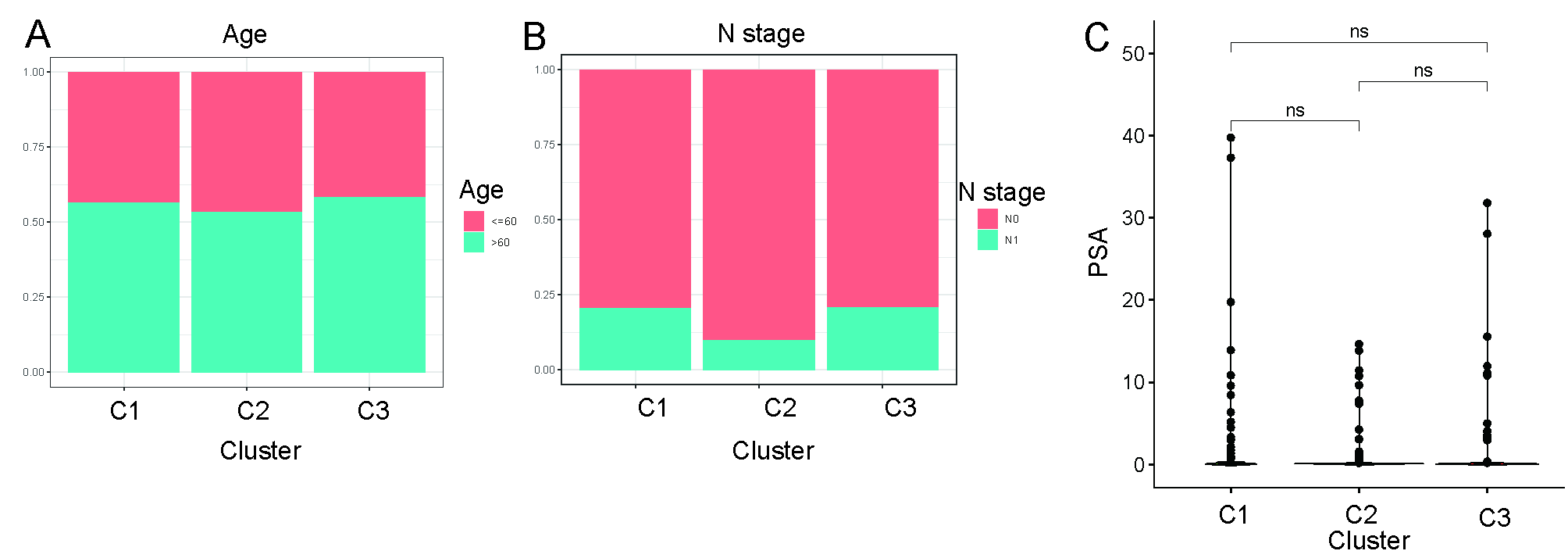

Supplement: Supplementary Figure 2 — Association between clinical characteristics and the metabolism-associated subtypes. Age (A) and Lymph Nodes (N) stage (B) for each metabolism-associated subtype in the TCGA cohort. (C) The pairwise comparison of the PSA between three subtypes. The P values are labeled above each boxplot with asterisks (ns represents no significance, *P <0.05, **P <0.01, ***P <0.001). [file Image_2.tif]

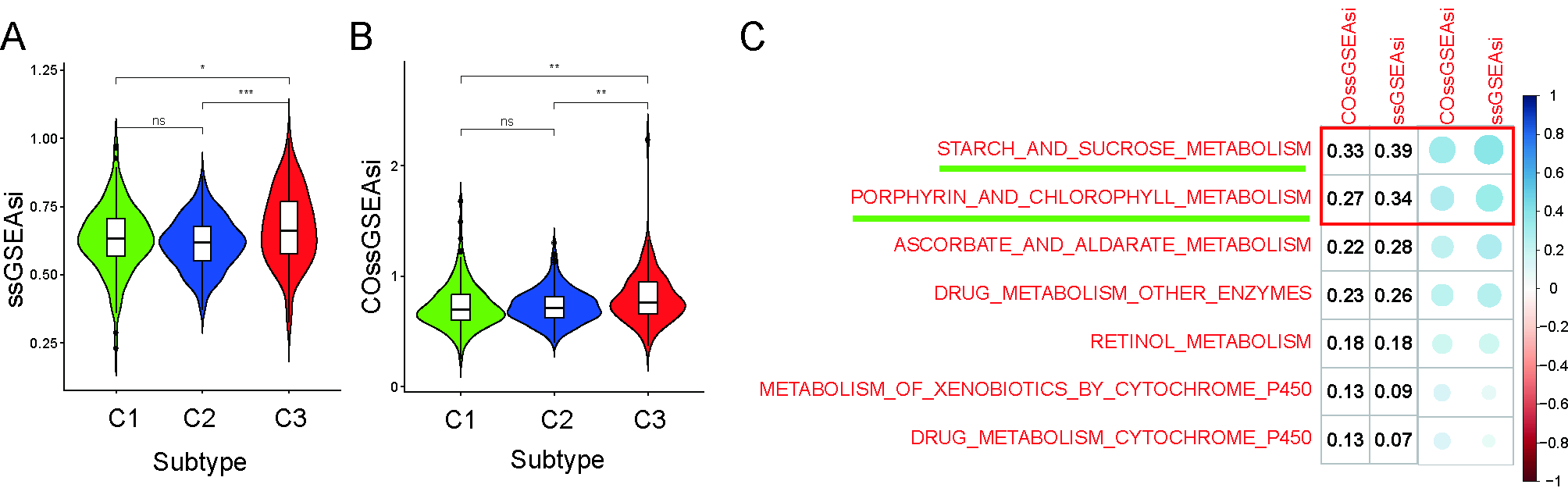

Supplement: Supplementary Figure 3 — Association between the stemness index and the metabolism-associated subtypes. The pairwise comparison of the ssGSEAsi (A) and COssGSEAsi (B) between three subtypes. The P values are labeled above each boxplot with asterisks (ns represents no significance, *P <0.05, **P <0.01, ***P <0.001). (C) The correlation analysis between specific metabolic pathways of C3 and ssGSEAsi in the TCGA cohort. [file Image_3.tif]

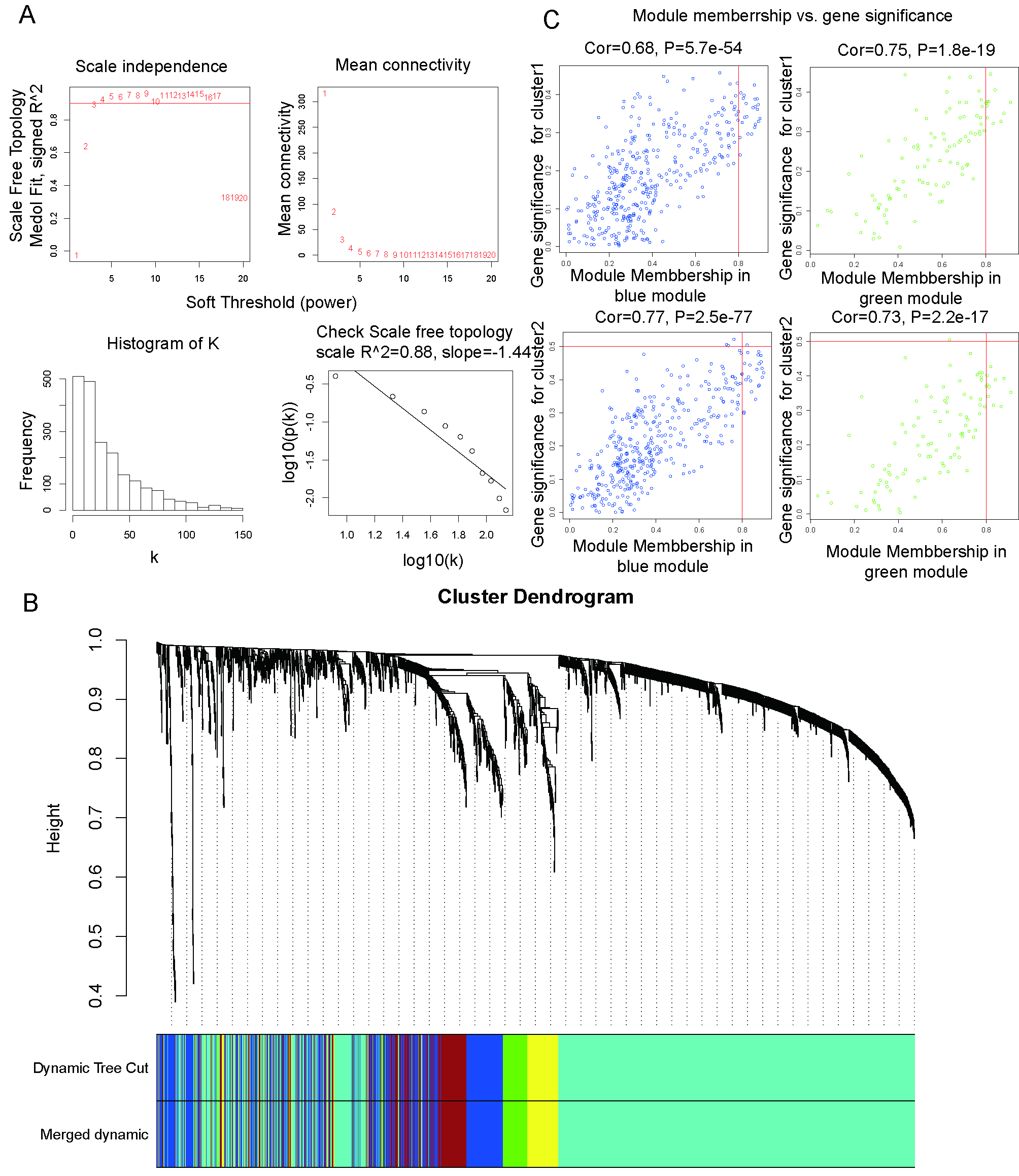

Supplement: Supplementary Figure 4 — The WCGNA analysis of metabolism-associated genes among three subtypes. (A) Analysis of the scale-free fit index and the mean connectivity for various soft-thresholding powers, and checking the scale-free topology when β = 3. K represents the logarithm of whole network connectivity, p(k) represents the logarithm of the corresponding frequency distribution. K is negatively correlated with p(k) (correlation coefficient = 0.88), which represents scale-free topology. (B) Identification of a co-expression module in PCa. The branches of the cluster dendrogram correspond to the five different gene modules. Each piece of the leaves on the cluster dendrogram corresponds to a gene. (C) Scatter plot of module eigengenes in the blue and green modules. [file Image_4.tif]

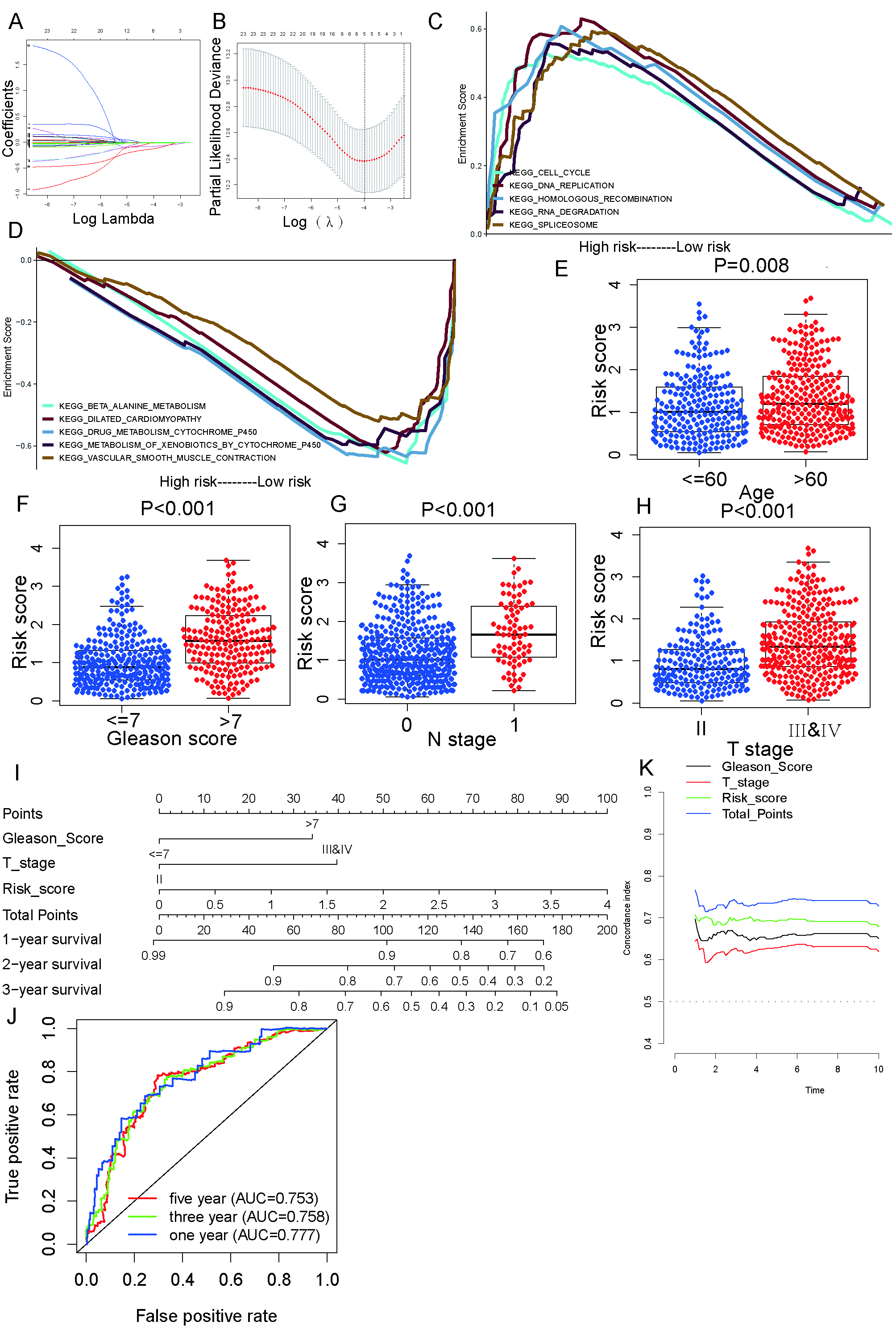

Supplement: Supplementary Figure 5 — Identification of risk model functions and development of the nomogram. (A, B) prognostic genes selected through Lasso regression. (C, D) Enrichment plots of the top five KEGG pathways in the high-risk score and low-risk score groups in PRAD. The relationship between the age (E)/Gleason score (F)/N stage (G)/T stage (H) and risk score in the TCGA cohort. (I) Nomogram for predicting the probability of 1, 3, and 5 disease-free survival times for PCa patients. (J) ROC analysis for nomogram in 1, 3, and 5 years. (K) The C-index analysis for clinical variates, risk model, and nomogram. [file Image_5.tif]

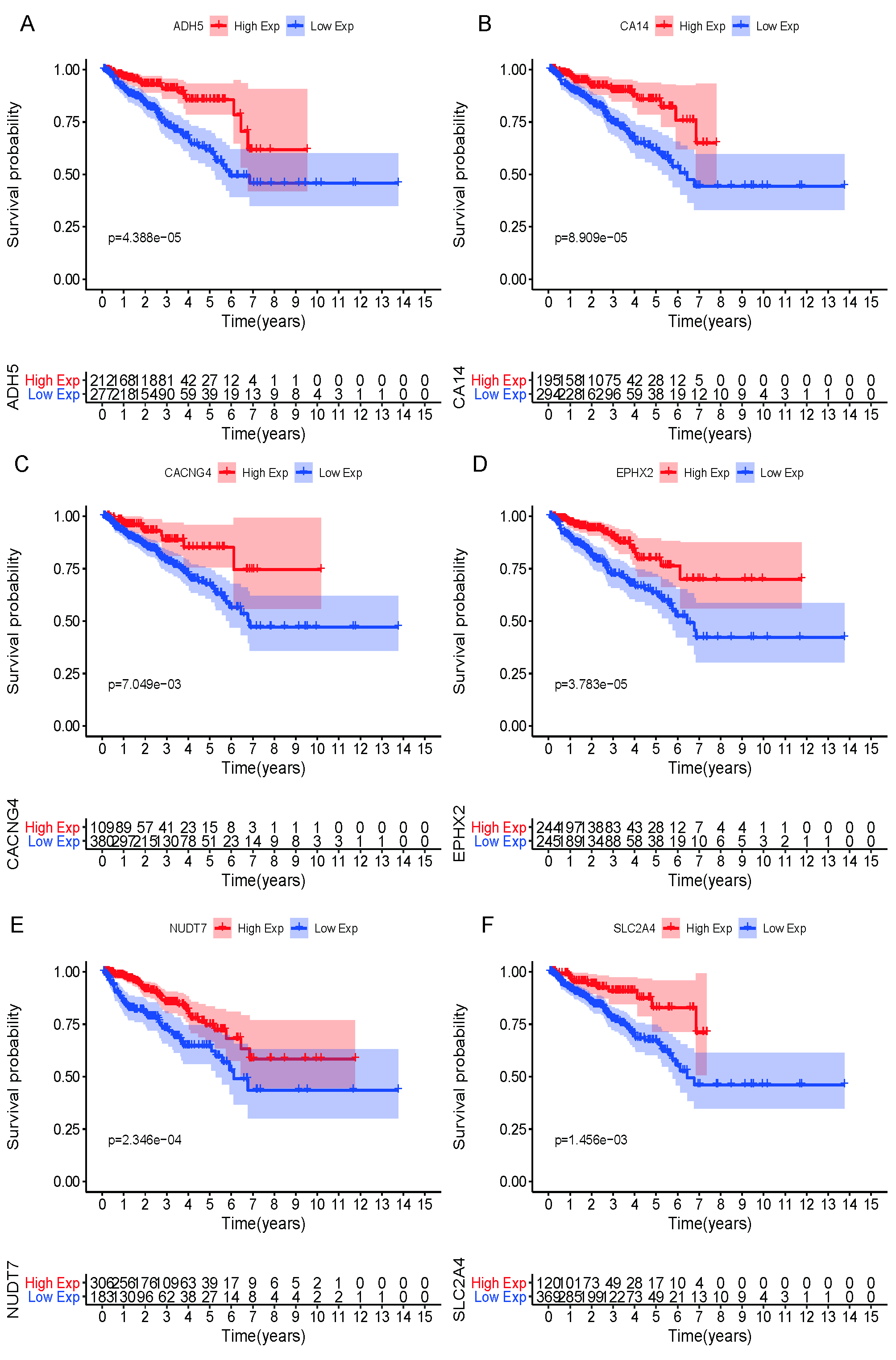

Supplement: Supplementary Figure 6 — The K-M survival analysis of six risk model genes. (A) ADH5. (B) CA14. (C) CACNG4. (D) EPHX2. (E) NUDT7. (F) SLC2A4. [file Image_6.tif]

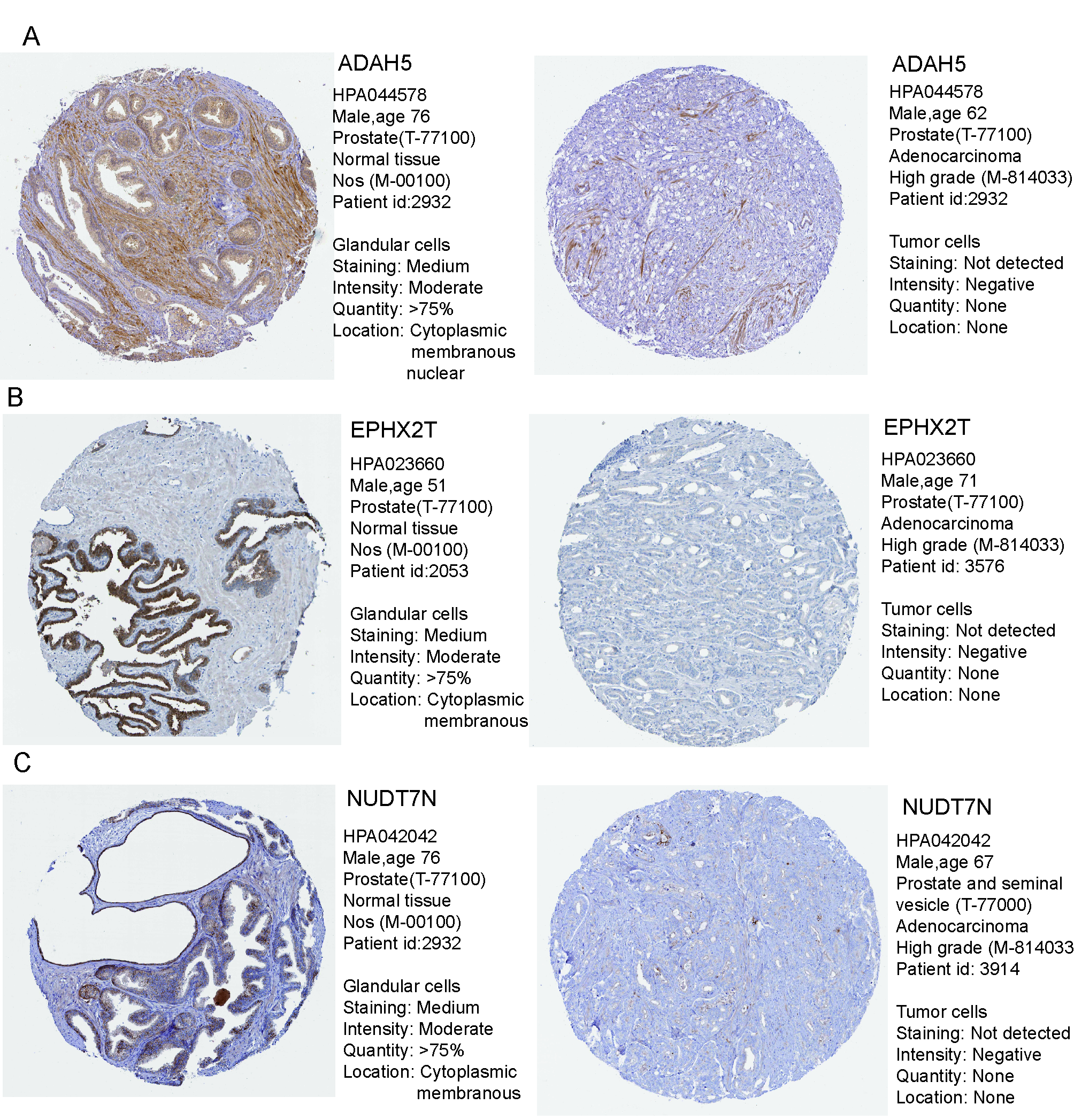

Supplement: Supplementary Figure 7 — The protein expression of three risk model genes in normal prostate tissues and PRAD tissues from the Human Protein Atlas (HPA) database. (A) ADAH5. (B) EPHX2T. (C) NUDT7N. [file Image_7.tif]
